# Shifting the focus to functioning: essential for achieving Sustainable Development Goal 3, inclusive Universal Health Coverage and supporting COVID-19 survivors

**DOI:** 10.1080/16549716.2021.1903214

**Published:** 2021-04-27

**Authors:** Dorothy Boggs, Sarah Polack, Hannah Kuper, Allen Foster

**Affiliations:** a International Centre for Evidence in Disability, London School of Hygiene & Tropical Medicine, London, UK; b International Centre for Eye Health, London School of Hygiene & Tropical Medicine, London, UK

**Keywords:** Functioning, population measurement, global health, rehabilitation, assistive technology

## Abstract

If Sustainable Developmental Goal 3 and Universal Health Coverage are to be achieved, functioning is a third health indicator which must be assessed and integrated into global health population-based metrics alongside mortality and morbidity. In this paper, we define functioning according to the International Classification of Functioning, Disability and Health (ICF) and present why functioning is important to measure, especially when considering the need for, and outcome of, rehabilitation and assistive technology. We discuss examples of tools that measure components of functioning through clinical assessment and self-report methodologies, and present the development of a comprehensive population level tool which aligns with the ICF and combines self-report and clinical measurement methods to measure functioning and the need for rehabilitation and AT. Throughout the paper a survivor of Coronavirus 2019 (COVID-19) is given as an example to illustrate functioning according to the ICF and how access to the interventions of rehabilitation and assistive technology might be of benefit to improve and optimise his/her functioning. We argue that the Global Health community must take action and ensure that the measurement of functioning is well established, accepted and integrated as the third health indicator following the COVID-19 pandemic.

## Background

Historically population-based metrics in Global Health have relied heavily on mortality and morbidity. These two health indicators have accumulated great importance and are used widely when assessing health within nations and populations. Though gaps still remain, mortality and morbidity data have led to the development of life-saving health interventions and are increasingly routinely measured in health systems. Morbidity is defined as having a disease or the amount of disease in a population, but what about the Global Health metrics *after* morbidity? As members of a population survive with health conditions, including communicable or non-communicable diseases, what indicator is available to measure their lived experiences of health throughout the life course?

The importance of these questions can be illustrated through the coronavirus disease 2019 (COVID-19). Though much is still unknown about COVID-19 and the recovery trajectory, it is increasingly clear that many COVID-19 survivors experience difficulties in functioning following both hospitalisation for severe acute disease and recovery from mild to moderate symptoms in home/community settings. Evidence suggests high physical, neuropsychological and social need, and that the most common post-COVID symptoms are fatigue, breathlessness and psychological distress, including depression, anxiety and PTSD [1]. Many COVID-19 survivors are experiencing these symptoms alongside several months of general deconditioning, leading to the now more common terminology of ‘Long COVID’; yet, the issue of Long COVID and the needs of survivors are not being identified and addressed [1–3].

## Functioning, the third indicator in population health

**Figure 1.**
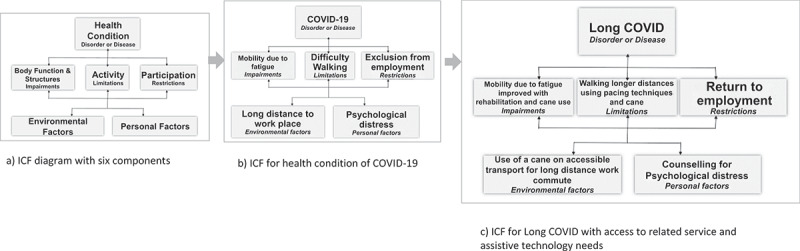
Example of International Classification of Functioning, Health and Disability (ICF) diagram for health condition of coronavirus disease 2019 (COVID-19) with access to related service and assistive technology needs [5]

An essential complementary third health indicator, ***functioning***, provides metrics about *how people are living in their daily lives* [4]. Functioning is defined as an umbrella term in the International Classification of Functioning, Disability and Health (ICF- Figure 1(a)) for body functions and structures, activities, and participation; it denotes the interaction between an individual (with a health condition) and his/her contextual factors (environmental and personal factors) [5]. Functioning is complex given it incorporates all of the six key ICF components and is incorporated in a supplementary section of the International Classification of Diseases eleventh revision (ICD-11) [6].

Figure 1(b) presents an example of functioning, using the ICF framework, as applied to a hypothetical COVID-19 survivor. In this example, a COVID-19 survivor experiences a mobility impairment due to high levels of fatigue and breathlessness resulting in poor endurance. He/she might experience difficulty walking long distances (activity restriction) preventing the survivor from getting to his/her office job (participation restriction) in the context of a long-distance commute involving both walking and public transport (environmental factor). These difficulties may also result in psychological distress (personal factor) which in turn may further limit participation in work.

## Why is measuring function important?

Functioning data are vital to understand the experiences of people with disabilities, older people and people living with chronic health conditions, non-communicable diseases, and communicable diseases with long term conditions, including COVID-19 survivors. More broadly, functioning is critical as the Global Health community aims to achieve Sustainable Development Goal (SDG) 3 ‘Ensure healthy lives and promote well-being for all at all ages’ and Universal Health Coverage (UHC) [7]; we cannot know if we are *actually* reaching the most vulnerable and marginalised populations if we don’t have accurate data on who they are, what they have difficulty doing, and how their daily lives could be improved?

These factors have become even more important given changing global health and demographic trends, and the increased numbers of people experiencing functional difficulties and disability [8,9]. Further, given functioning is environmental and personal context dependent, population-based functioning needs will change over time as populations age and contexts change and adapt. Functioning indicators could enable more responsive measurement and monitoring of specific needs within contexts and settings. For example, Disability-Adjusted Life Years (DALYs), a widely used population health disability measurement, are primarily based upon the impact of living with a health condition’s impairment (i.e. body function and structure component of the ICF) that is associated with certain functional limitations. DALYs are not sensitive enough to be able to measure people’s overall functioning resulting from either changes over time (with or without interventions) or interactions with other components of the ICF, such as personal and environmental factors, recognising that functioning can change even if an underlying ‘health condition’ does not [10,11]. Therefore, identifying, measuring and monitoring population-based functioning incorporating *all* ICF components will be key for advancing the agenda for this indicator. Functioning data are important for informing evidence-based health and social rights-based policies, planning services and identifying appropriate interventions that can support populations to live more holistic and complete lives. This data will provide information about an individual’s health in a more comprehensive way, which will in turn support broader cross-sectoral interventions.

Rehabilitation and assistive technology (AT) are two inter-related sectors that rely on functional assessment to identify appropriate interventions to optimise functioning and independence. Comprehensive data on functioning at the population-level are key for identifying need/unmet need for rehabilitation and AT. However, as both sectors advance their global agendas, these data are lacking in many areas of the world, constraining the effective planning and provision of these services [12,13]. Estimates that are available, such as the recent WHO estimates that 2.4 billion or one in three people are in need of rehabilitation services, are often based upon gross estimates of Global Burden of Disease data [14]. These need to be advanced with more accurate disaggregated measurement.

## How to measure: functioning, rehabilitation and AT?

Given the importance of data on functioning, how can it be measured?

As summarised in Table 1, different methods are used to assess functioning, and/or rehabilitation or AT needs at the population-level (e.g. through surveys). However, most of them capture only one or a sub-set of the six ICF components.

**Table 1.** Examples of four tools that measure components of the International Classification of Functioning, Health and Disability (ICF) [5]

Measurement tool example	Tool method	Number of ICF component/s	Specific ICF component/s measured	Description
RAAB [16]	Clinical measurement	1	Impairment	Objectively measures distance and near visual impairment (VI) and assesses cause. RAAB 7 integrates Peek acuity which assesses visual acuity by a non-specialist using a smartphone.
Washington Group on Disability question sets [17]	Self-report	1	Activity limitations	*Short question module*: six questions for participants ≥5 years old measuring six core functional domains- seeing, hearing, walking, cognition, self-care, and communication. *Extended question module*: 29 questions for participants ≥5 years old including short set module plus upper body functioning, psychosocial difficulties, pain and fatigue, and additional information in certain domains of functioning both with and without the use of assistive technology/aids. *Child functioning module*: two question sets for participants between 2–4 years old and 5–17 years assessing functional difficulties in different domains including hearing, vision, communication/comprehension, learning, mobility and emotions.
WHO Model Disability Survey [18]	Self-report	6	Health condition; body function; activity limitations; participation restrictions; environment and personal factors	Self-reported questionnaire that asks people what they do, or do not do, in their daily lives focusing on functioning in multiple domains well-aligned with the ICF and a series of questions regarding domain-specific and participation-specific health service, rehabilitation and AT use.
WHO GATE’s rapid Assistive Technology Assessment [19]	Self-report	3	Activity limitations; participation restrictions; environment	Self-reported questionnaire that assesses participants’ need, unmet need and access to AT using adapted Washington Group Short Set as initial screening and images alongside each assistive product.

Two of the most commonly used approaches are clinical measurement and self-report; however, they produce inconsistent results and typically remain siloed, and do not provide holistic cross-ICF component measurement [15]. Clinical measurement typically focuses solely upon body structure and function. Clinical impairment-based assessments are important for identifying select health-related service needs (e.g. surgical, medical and some ‘correctable’ impairment service referrals such as spectacles for refractive error), but they do not capture broader aspects of a person’s functioning (e.g. activities, participation and context) as defined by the ICF [15]. For example, the Rapid Assessment of Avoidable Blindness (RAAB) [16] is a widely used impairment survey method which includes visual acuity assessment and eye examinations to identify visual impairment and likely ‘cause’, such as cataracts and refractive error. Referrals to surgical, medical and vision services are made based on this information.

Self-reported functioning measures are cheaper and easier to administer than clinical measures. The Washington Group on Disability question sets ask about difficulty completing activities, such as the Short set which focuses upon activities in six domains (seeing, hearing, walking, remembering, understanding and self-care) alongside select AT use [17]. These tools are short to administer and widely used internationally. However, they primarily focus on the activity limitation component of the ICF only. The self-reported WHO Model Disability Survey [18] incorporates all six ICF components to assess broader health and social needs, including rehabilitation and AT use, with the brief version recommended in the ICD-11 functioning assessment supplementary section, and the WHO rapid Assistive Technology Assessment focuses upon self-reported activity, participation and environment components to assess AT use and need [19]. However, evidence suggests that self-report alone is unreliable and can either over- or under-estimate functioning difficulties and related needs [15]. A comprehensive functional assessment approach which incorporates all the ICF components is lacking. This is needed to inform rehabilitation and AT service needs, as well as other interventions.

Returning to the COVID-19 example, identifying long term effects, such as vocal cord damage from invasive ventilator use, and associated functional difficulties with COVID-19 and its variants will require functional screening and measurement tools across multiple domains at both individual and population levels. It also will be important to ensure disaggregation of these data by key characteristics, such as age, race, ethnicity, gender, disability and other socio-demographic variables as well as qualitative methods to explore lived experience in more depth. Further, managing functional needs will require i) person-centred care; ii) a continuum of care from clinicians to community workers, and; iii) uptake of referrals to rehabilitation and AT interventions from acute to community health settings either virtually or face-to face [3,20]. In Figure 1(c), for the same person, access to rehabilitation services including counselling and the use of a single-point cane on accessible transport could facilitate participation in his/her job.

The ‘Post-COVID-19 Functional Status (PCFS) Scale’ is a self-report screening tool designed for telephone administration to assess the spectrum of functional outcomes following COVID-19 and track progress over time [21]. However, there is a need for more comprehensive tools which integrate clinical impairment assessment as well as other ICF components to assess functioning in different domains. This will be important to better understand functioning and associated need for rehabilitation/AT services and to highlight an important treatment gap [20]. This assessment method could then be applied more broadly to other communicable and non-communicable diseases, injuries and health conditions, and be used for planning and advocating for health system strengthening of these interventions.

A gap remains for a comprehensive tool, not just specific to COVID-19, which can be used at the population level to measure functioning and the need for rehabilitation and AT. In the AT2030 research funded by UK Aid, a functional needs assessment tool is being developed and tested which combines self-report and clinical measurement methods incorporating all ICF components [15]. Maintaining a people-centred approach is fundamental. Therefore, functioning data will be collected to capture the individual’s impairment, participation, activities and environmental and personal contexts across the functional domains of vision, hearing, mobility, communication, cognition, self-care and mental health.

## Action: build back better with an inclusive focus on functioning

As the world grapples to ‘build back better’ following the COVID-19 pandemic and at the same time advance the SDG and UHC agendas, it is important to remember the SDGs’ tagline ‘leave no one behind’. To do this it is essential to ensure that the measurement of functioning is well established, accepted and integrated as the third health indicator. Increased attention is needed to ensure improved clarity, consistency and understanding of its definition and measurement. Development and application of population-based assessment tools which incorporate all components of the ICF will be important for generating comprehensive and comparable data on functioning needed to inform rehabilitation and AT, as well as other interventions/services. To action this, the Global Health community is encouraged to lead a shift of terminology and mindset from focusing on ‘mortality’ and ‘morbidity’ to equally include ‘***functioning.’*** This resultant scaling up of the measurement of functioning will enable us to inclusively build back better, improving health and wellbeing for *all*.

## Paper context

Alongside mortality and morbidity, functioning is a third health indicator which must be assessed and integrated into global health population-based metrics. This paper defines functioning, presents measurement options and highlights the importance of functioning when considering the need for, and outcome of, rehabilitation and assistive technology following a health condition illustrated by the example of COVID-19. Increased attention is needed to ensure improved clarity, consistency and understanding of the definition and measurement of functioning.
